# Dynamic incorporation of prior knowledge from multiple domains in biomarker discovery

**DOI:** 10.1186/s12859-020-3344-x

**Published:** 2020-03-11

**Authors:** Xin Guan, George Runger, Li Liu

**Affiliations:** 1 College of Health Solutions, Arizona State University, Phoenix, AZ 85004 USA; 2 Intel Corporation, Chandler, AZ 85226 USA; 3 Biodesign Institute, Arizona State University, Tempe, AZ 85287 USA; 4 Department of Neurology, Mayo Clinic, Scottsdale, AZ 85259 USA

**Keywords:** Biomarker discovery, Domain knowledge, Feature selection, Regularized random forest

## Abstract

**Background:**

In biomarker discovery, applying domain knowledge is an effective approach to eliminating false positive features, prioritizing functionally impactful markers and facilitating the interpretation of predictive signatures. Several computational methods have been developed that formulate the knowledge-based biomarker discovery as a feature selection problem guided by prior information. These methods often require that prior information is encoded as a single score and the algorithms are optimized for biological knowledge of a specific type. However, in practice, domain knowledge from diverse resources can provide complementary information. But no current methods can integrate heterogeneous prior information for biomarker discovery. To address this problem, we developed the Know-GRRF (know-guided regularized random forest) method that enables dynamic incorporation of domain knowledge from multiple disciplines to guide feature selection.

**Results:**

Know-GRRF embeds domain knowledge in a regularized random forest framework. It combines prior information from multiple domains in a linear model to derive a composite score, which, together with other tuning parameters, controls the regularization of the random forests model. Know-GRRF concurrently optimizes the weight given to each type of domain knowledge and other tuning parameters to minimize the AIC of out-of-bag predictions. The objective is to select a compact feature subset that has a high discriminative power and strong functional relevance to the biological phenotype.

Via rigorous simulations, we show that Know-GRRF guided by multiple-domain prior information outperforms feature selection methods guided by single-domain prior information or no prior information. We then applied Known-GRRF to a real-world study to identify prognostic biomarkers of prostate cancers. We evaluated the combination of cancer-related gene annotations, evolutionary conservation and pre-computed statistical scores as the prior knowledge to assemble a panel of biomarkers. We discovered a compact set of biomarkers with significant improvements on prediction accuracies.

**Conclusions:**

Know-GRRF is a powerful novel method to incorporate knowledge from multiple domains for feature selection. It has a broad range of applications in biomarker discoveries. We implemented this method and released a KnowGRRF package in the R/CRAN archive.

## Background

Biomarker discovery aims to identify a concise molecular signature of a biological phenotype from among a large number of features. To facilitate this process, data-driven feature selection methods have been widely employed that prioritize features based on their discriminative power. However, the low signal-to-noise ratio in large-scale omics data and the complex dependencies among features pose a grand challenge to data-driven methods. Without additional constraints, these methods often produce suboptimal solutions that include many false positive markers and overlook functionally impactful features. Consequently, predictive models built on these features may suffer from under-fitting or over-fitting problems [1–3].

One solution to these issues is integrating multi-omics data that characterize different aspects of a complex biological system. Several computational methods have been developed for this purpose (reviewed in [4]). Another solution is to combine expert knowledge with statistical analysis [5–8]. The most straightforward and common practice applies domain knowledge as a post hoc filter by ranking statistically significant features based on functional annotations from external databases [9, 10]. A more sophisticated approach involves systematic evaluations of biomarkers on their discriminative power and biological relevance. For example, Peterson et al. considered gene-network as informative prior and performed a joint Bayesian variable and graph selection in regression models [11]. Park et al. proposed a *l*_1_-regularized linear regression model that prioritizes cancer genes showing dependence of copy number alterations on expression levels [12]. Although these methods perform well in specific domains, the feasibility of using these methods to incorporate knowledge from other domains remains unclear. Meanwhile, annotations from diverse resources likely provide complementary information. In a study of cancer prognostic biomarkers, Liu et al. showed that a composite score of evolutionary conservation and pre-computed statistical *p* values was more informative than individual scores when used as weights in regularized logistic regressions [7]. Given the availability of diverse functional annotations, a generalizable approach that can evaluate domain knowledge from heterogeneous resources and automatically determine the optimal combination for guided feature selection is highly desirable.

Previously, we developed the know-guided regularized random forest (Know-GRRF) algorithm that is a generalized form of regularized random forests (RRF) to enable the incorporation of prior information in feature selection [13]. Know-GRRF achieves regularization by introducing a penalty coefficient for each feature that is computed from a user-specified score (i.e., prior) and several system-tuned parameters. In this study, we extended the Know-GRRF algorithm to allow each feature to be associated with multiple priors. Specifically, Know-GRRF derives a linear model to combine multiple priors into a composite score. In this linear model, the contribution of each prior to the composite score is determined via maximum likelihood optimization, which is coupled with the optimization of other tuning parameters to minimize the Akaike’s information criterion (AIC) of out-of-bag (OOB) predictions [14, 15]. In various simulated scenarios, we demonstrated that integrating multiple prior information using Know-GRRF significantly improves feature selection accuracies. In a real-world application, we illustrated that Know-GRRF effectively aggregated knowledge from multiple domains to facilitate the discovery of prognostic biomarkers of prostate cancers.

## Results

### The Know-GRRF METHOD

**Fig. 1:**
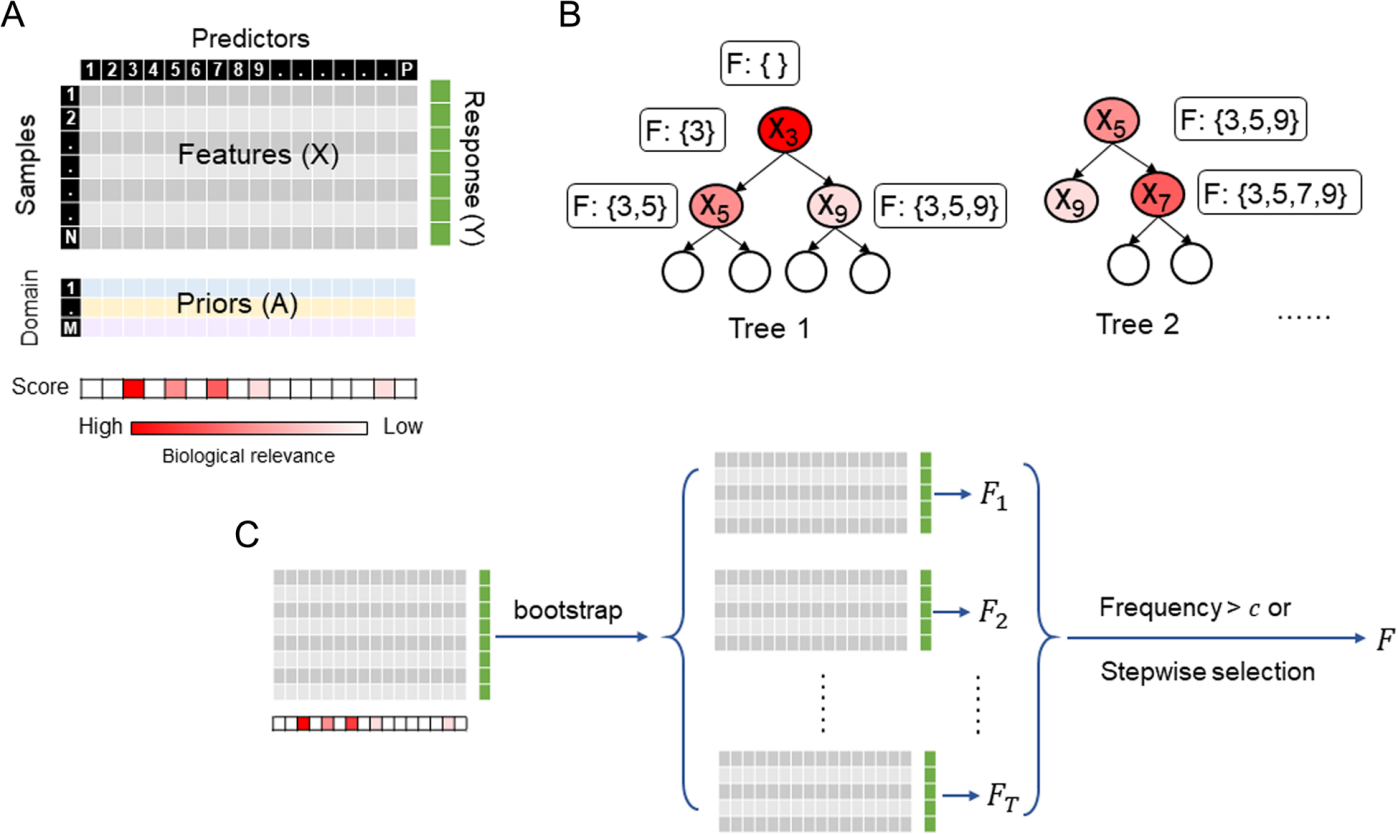
Schematic representation of the Know-GRRF method. (**a**) The data structure. The feature matrix *X* contains the observed values of *P* predictors of *N* samples. The prior matrix *A* contains functional measures of each predictor from *M* domains. These functional measures are combined in a linear model to derive a *score* representing the biological relevance of predictors. The vector *Y* contains the observed response values of the samples. (**b**) The feature selection component. Non-leaf nodes are marked with the splitting features and colored by the corresponding biological relevance. Know-GRRF starts with an empty feature set *F*. In tree 1, three features (*X*_3_, *X*_5_ and *X*_9_) are sequentially added to *F* based on information gains weighted by biological relevance. In tree 2, because *X*_5_ and *X*_9_ are already members of *F*, they are selected based on information gains only. Because *X*_7_ is not a member of *F*, it is selected based on information gain weighted by biological relevance. (**cmsubsup**) The stability selection component. Know-GRRF first optimizes the tuning parameters on the complete dataset. It then uses bootstrapped samples to select features. After *T* iterations, features selected in more than a user-define frequency cutoff *c* are aggregated and constitute the final feature set. Alternatively, Know-GRRF can use stepwise selection to derive the final feature set

We show the schematic representation of the data structure and the algorithm of Know-GRRF in Fig. 1. The data set consists of *N* samples, each measured on one response variable and *P* predictor variables. Each predictor is associated with a set of priors from *M* domains (Fig. 1a). We denote *Y*_*i*_ as the observed response value of sample *i* where *i* = 1, …, *N*. We denote ${X}_i^j$ as the observed value of predictor *j* of sample *i* where *j* = 1, …, *P*. We denote ${A}_d^j$ as the prior relevance score on predictor *j* from domain *d* where *d* = 1, …, *M*. A linear model combines priors from all domains into a composite *score*_*j*_ that represents the biological relevance of predictor *j*. The objective of Know-GRRF is to model the relationship between *X* and *Y* with a compact feature subset such that the biological relevance of selected features is maximized and the loss of predictive accuracy is minimized. To achieve this goal, the core algorithm of Know-GRRF consists of two components.

The first component selects features using RRF guided by prior information from multiple domains. As in the ordinary random forests algorithm [15], Know-GRRF learns multiple decision trees (from bootstrapped samples) to model the relationship between *X* and *Y*. At a splitting node *v* of a tree, a predictor *j* is evaluated based on the regularized information gain as

1 $$ {Gain}_R\left({X}^j,v\right)=\left\{\begin{array}{c}\ {\lambda}_j Gain\left({X}^j,v\right)\ j\notin F\\ {} Gain\left({X}^j,v\right)\ j\in F\end{array}\right. $$

where *λ*_*j*_ ∈ [0, 1] is a penalty coefficient, *Gain* (*X*^*j*^, *v*) is the Gini information gain [15], and *F* is a set of predictors selected in previous nodes. Here *F* begins as an empty set. At each splitting node, the selected predictor is added to *F* (Fig. 1b). If predictor *j* is not selected in previous nodes, *λ*_*j*_ penalizes it by reducing its information gain. The predictor with the highest *Gain*_*R*_(*X*_*i*_, *v*) is then selected and added to the feature set *F*. Know-GRRF defines the penalty coefficient *λ*_*j*_ as

2 $$ {\lambda}_j={score_j}^{\delta } $$

where *score*_*j*_ ∈ [0, 1] is the biological relevance of predictor *j*, and *δ* ∈ [0, ∞] is the tuning parameter. A higher value of *score*_*i*_ indicates higher biological relevance. If multiple prior information is available for a predictor, *score*_*j*_ is computed as

3 $$ {score}_j=\frac{\sum_{d=1}^M{\beta}_d{A}_d^j}{\underset{j}{\max }{\sum}_{d=1}^M{\beta}_d{A}_d^j} $$

where *β*_*d*_ ∈ [0, 1] is the weight given to the prior information from domain *d*.

The objective of Know-GRRF is to select a compact collection of predictors that constitute *F* without loss of predictive information about *Y*. We use AIC of OOB predictions as the loss function

4 $$ AIC=2k-2\ln \left(\hat{L}\right) $$

where *k* is the number of predictors in *F* and $\ln \left(\hat{L}\right)$ is a is goodness-of-fit measure [14]. Know-GRRF then uses the BFGS quasi-Newton method [16] to optimize the tuning parameter *δ* and the prior information weight *β* within a user-specified range to minimize AIC.

The second component performs a stability selection that chooses a set of reliable predictors across multiple runs (Fig. 1c). In RRF, a challenge is the large variance from run to run due to the randomness of bootstrapping. To address this problem, after obtaining the optimal values of *δ* and *β*, we perform a stability selection. Specifically, with the optimal *δ* and *β* values, we build Know-GRRF models *t* times, each time using 90% of randomly selected samples, which returns *t* sets of selected predictors. To derive the final feature set, Know-GRRF offers two options. For the first option, users can specify a frequency cutoff *c* ∈ [0, 1] such that predictors selected in greater than or equal to *ct* iterations constitute the final solution. The default values of *t* and *c* are 10 and 0.5, respectively. For the second option, features selected in any of the *t* iterations are aggregated and are subject to a stepwise selection procedure to minimize the AIC, which then produces the final feature set.

### Performance on simulated data

We have previously compared Know-GRRF with other RRF-based feature selection methods and demonstrated the superior performance of Know-GRRF [13]. In this study, we focused on evaluating the influence of multiple domain knowledge on the feature selection results. We first present simulation results and then applied Know-GRRF to a gene expression study of metastatic prostate cancers to discover prognostic biomarkers.

#### Datasets

We simulated three scenarios with varying levels of complexity, including linear relationship, higher-order relationship and interaction. For each scenario, we generated 200 samples. Each sample was measured on 100 features (i.e., *j* = 1, …, 100). Each feature follows a normal distribution ${X}^j\sim \mathcal{N}\left(\mu, {\sigma}^2\right)$ with the mean and standard deviation drawn from continuous uniform distributions as *μ*~*U*(0, 5) and *σ*~*U*(1, 2). We used the first 10 features as true predictors and computed the response values (*Y*) following the predefined equations (Table 1). In scenario 1, informative features are independent and their linear combinations determined the response. In scenario 2, the second order product of feature *X*^10^ contributed to the response. In scenario 3, features *X*^9^ and *X*^10^ had interactions. The remaining features were uninformative (i.e. false predictors). For regression tasks, we used the original values of *Y*. For classification tasks, we dichotomized *Y* to a binary vector with the median value as the cutoff.

**Table 1:** True Relationship in Simulated Scenarios

Scenario	Relationships
1. Linear	*Y* = 1.6 + 2.6*X*^1^ + 1.7*X*^2^ + 1.3*X*^3^ + 1.4*X*^4^ − 2.5*X*^5^ + 1.9*X*^6^ + 2.3*X*^7^ + 4.5*X*^8^ − 0.3*X*^9^ + 0.4*X*^10^
2. Higher order	*Y* = 4.3 + 2.1*X*^1^ + 2.1*X*^2^ + 3.4*X*^3^ + 2.6*X*^4^ + 3.2*X*^5^ + 5.9*X*^6^ + 1.5*X*^7^ + 1.1*X*^8^ − 1.2*X*^9^ + 2.6*X*^10^*X*^10^
3. Interaction	*Y* = 2.3 + 2.6*X*^1^ + 3.9*X*^2^ + 2.8*X*^3^ + 5.5*X*^4^ + 3.3*X*^5^ − 2.5*X*^6^ + 1.0*X*^7^ + 2.5*X*^8^ + 1.3*X*^9^*X*^10^

Superscripts indicate the indices of feature X

We simulated complementary prior knowledge from two domains. Specifically, we used high relevance scores sampled from a normal distribution $\mathcal{N}\left(5,1\right)$ to indicate informative features, and low relevance scores sampled from a normal distribution $\mathcal{N}\left(0,1\right)$ to indicate uninformative features. In domain one, features 1 to 5 received high relevance scores. In domain two, features 6 to 10 received high relevance scores. The other features received low relevance score. Negative scores were reset to zero.

We applied Know-GRRF to identify informative features with priors only from the first domain, with priors only from the second domain, and with priors from both domains. As negative controls, we applied RFF [17] and Lasso [18] to selecting features with no prior information and chose their regularization parameters (*γ* and *λ*, respectively) corresponding to the highest accuracies via grid searches (details in Methods). To quantify the similarity between two feature sets (i.e. simulated true predictor set *F*_1_ vs. method selected set *F*_2_), we computed the Jaccard Index (*JI* = |*F*_1_ ∩ *F*_2_|/|*F*_1_ ∪ *F*_2_|), true positive rate (*TPR* = |*F*_1_ ∩ *F*_2_|/| *F*_1_|) and false positive rate (*FPR* = |*F*_2_ − *F*_1_|/| *F*_2_|). We also reported false negatives (FN) that are true informative features not selected by a method.

#### Classification tasks

For the two-class classification tasks in different scenarios, we summarized the performance of Know-GRRF with priors from different domains and the performance of RRF and Lasso with no priors in Table 2.

**Table 2:** Methods Comparison in Two-Class Classification Tasks

Method	Lasso	RRF	Know-GRRF					
			prior 1		prior 2		prior both	
			Freq>50%	Stepwise	Freq>50%	Stepwise	Freq>50%	Stepwise
Scenario 1								
JI	0.26	0.18	0.40	0.40	0.50	0.33	0.80^a^	0.80^a^
TPR	0.90	0.30	0.40	0.40	0.50	0.40	0.80	0.80
FPR	0.27	0.08	0	0	0	0.03	0	0
FN	10	1, 2, 3, 4, 7, 9, 10	4, 6, 7, 8, 9, 10	4, 6, 7, 8, 9, 10	1, 2, 3, 4, 5	1, 2, 3, 4, 5, 10	2, 4	4, 10
Scenario 2								
JI	0.38	0.19	0.47	0.55	0.50	0.30	0.60	0.80^a^
TPR	0.80	0.40	0.70	0.60	0.50	0.30	0.60	0.80
FPR	0.12	0.12	0.06	0.01	0	0	0	0
FN	3, 9	1, 2, 4, 7, 8, 9	7, 8, 9	4, 7, 8, 9	1, 2, 3, 4, 5	1, 2, 3, 4, 7, 8, 9	1, 3, 5, 9	1, 9
Scenario 3								
JI	0.31	0.27	0.40	0.27	0.50	0.30	0.50	0.90^a^
TPR	1.00	0.40	0.40	0.30	0.50	0.30	0.50	0.90
FPR	0.24	0.06	0	0.01	0	0	0	0
FN		1, 3, 5, 6, 7, 8	1, 6, 7, 8, 9, 10	1, 3, 6, 7, 8, 9, 10	1, 2, 3, 4, 5	1, 2, 3, 4, 7, 8, 9	1, 3, 4, 5, 7	3

^a^indicates the best JI value in each scenario

As expected, Know-GRRF consistently outperformed RRF and Lasso. We observed the greatest improvement in scenario 1 where Know-GRRF using priors from both domains had a large JI value of 0.80 and RRF and Lasso had small JI values of 0.18 and 0.26, respectively. In all scenarios, Know-GRRF using priors from both domains outperformed Know-GRRF using single-domain priors. This is not surprising because we simulated complementary priors from the two domains. However, it was worth noting that Know-GRRF using priors from a single domain identified some of the informative features even if they were not indicated as relevant by the priors. This implicated that both the discriminative power and the prior information were considered when selecting features.

Know-GRRF provides two options in the stability selection step to construct the final feature set. The first option uses selection frequency > 50% as the criteria and the second option uses stepwise selection to minimize the AIC value. The simulation results showed that these two options were complementary to each other if priors from a single domains was used. When priors from both domains were used, the stepwise selection approach had a better performance.

#### Regression tasks

We summarized the performance of Know-GRRF and RRF in Table 3. Except in scenario 1, Know-GRRF using priors from both domains had the highest JI value in all other scenarios. The superior performance of Lasso in scenario 1 was expected because Lasso is specifically optimized for first-order linear regressions with no interactions. Furthermore, in regression tasks, the TPR of Know-GRRF was significantly higher than in classification tasks (mean TPR = 0.73 vs. 0.51, paired two-sided t test *p*-value< 10^− 4^); and the FPR was also significantly higher (mean FPR = 0.006 vs. 0.10, paired two-sided t test p-value<10^− 7^). This can be explained by the trade-off between sensitivity and specificity, although the objective of Know-GRRF is to minimize the AIC value instead of the AUC value of an ROC curve. We also noticed that prior information on features with higher-order effects (feature 10 in scenario 2) or interactions (features 9 and 10 in scenario 3) were important. If the priors indicated these features were relevant, Know-GRRF could successfully identify these features. Otherwise, as previously reported [13, 19], it is a challenging task for Know-GRRF and other methods to detect interactions in the absence of main effects.

**Table 3:** Methods Comparison in Regression Tasks

Method	Lasso	RRF	Know-GRRF					
			Prior 1		Prior 2		Prior 3	
			Freq>50%	Stepwise	Freq>50%	Stepwise	Freq>50%	Stepwise
Scenario 1								
JI	0.91^a^	0.07	0.28	0.54	0.33	0.54	0.56	0.48
TPR	1.00	0.20	0.70	0.70	0.70	0.70	1.00	1.00
FPR	0.01	0.22	0.17	0.03	0.12	0.03	0.09	0.12
FN	–	2, 3, 4, 5, 6, 9, 10	6, 7, 10	6, 7, 10	2, 3, 4	2, 3, 4	–	–
Scenario 2								
JI	0.09	0.11	0.25	0.28	0.26	0.47	0.63^a^	0.53
TPR	0.10	0.30	0.70	0.50	0.60	0.80	1.00	1.00
FPR	0.01	0.19	0.20	0.09	0.14	0.08	0.07	0.10
FN	2, 3, 4, 5, 6, 7, 8, 9, 10	1, 2, 3, 4, 7, 8, 9	6, 8, 9	4, 6, 7, 8, 9	1, 2, 3, 4	3, 4	–	–
Scenario 3								
JI	0.67	0.19	0.29	0.57	0.26	0.47	0.56	0.67^a^
TPR	1.00	0.50	0.70	0.80	0.60	0.80	1.00	1.00
FPR	0.06	0.18	0.16	0.04	0.14	0.08	0.09	0.06
FN	–	1, 3, 5, 7, 8	7, 8, 10	7, 8	1, 2, 3, 5	1, 3	–	–

^a^indicates the best JI value in each scenario

### Application to discovering prognostic biomarkers for prostate cancers

We applied Know-GRRF, RFF and Lasso to a biomarker study to **discover** gene expression signatures that are predictive of metastasis of prostate cancers in 5 years [20]. In these analyses, these three methods accessed the same information on patient samples and gene expressions. However, RFF and Lasso were not capable of incorporating prior information of genes while Know-GRRF was tested on single-domain priors and multi-domain priors.

#### Dataset and pre-processing

This data set consisted of expression levels of 1021 genes in two cohorts of patients who were diagnosed with prostate cancer and received prostatectomy [20]. One cohort consisted of 201 patients showing no evidence of disease progression. The other cohort consisted of 200 patients who had metastatic recurrence within 5 years. Because none of these patients had increased level of prostate-specific antigen (PSA), novel biomarkers were needed to monitor the disease progression.

We downloaded the dataset from the NCBI GEO database (accession number: GSE10645). Using z-transformation, we normalized the expression levels of each gene to have a distribution with a mean of 0 and a standard deviation of 1. We then split the dataset into a training set that consisted of 360 randomly selected patients (181 with metastasis and 179 in remission) and a testing set that consisted of the remaining 41 samples (19 with metastasis and 22 in remission). For each gene, we performed two-sided Student t test to compare the expression level between the metastasis cohort and the remission cohort using the training data. We kept 251 genes with *p*-value < 0.01 as candidate biomarkers for Know-GRRF analysis.

#### Domain knowledge

We defined three types of prior information, each from a different domain. The first type of prior information was based on 526 genes that have been previously associated with prostate cancer aggressiveness [20] (see Methods for details). Among the 251 genes passing t-test, 169 genes were in this list and were assigned a prior score of 10 (cancer gene prior). Genes not in this list received a prior score of 1. The second type of prior information was based on evolutionary conservation. For each gene *g*, We computed the evolutionary rate (*R*_*g*_) using multiple alignments of 46 vertebrate genomes [21]. Because functionally essential genes are more conserved than non-essential genes [22], we defined a conservation prior score as 1/*R*_*g*_ (Consv. prior). The third type of prior information (VI prior) was based on the variable importance produced by RRF. We scaled all prior scores to a range between of 0 and 10 (see Methods for details).

#### Identify biomarkers using know-GRRF, RRF and lasso

We formulated biomarker discovery in this dataset as a two-class classification task. The metastasis status is the response variable (1 for metastasis and 0 for remission). Each gene represents a feature. We then applied Know-GRRF to identifying informative genes using the training dataset. We tested Know-GRRF with prior knowledge from a single domain and using prior knowledge from all thee domains. In each case, Know-GRRF optimized the values of *δ* and *β* concurrently to minimize the AIC in training data. In the stability selection step, because the frequency-based option and the stepwise selection option are complementary to each other, we used both options and took the union of the selected genes as the final set. We also applied RRF that uses no prior information. RRF requires a user-specified regularization parameter (*γ* ∈ [0, 1]). To determine the best value of *γ*, we performed grid searching with 20 values equally spaced between 0 and 1. Because the highest prediction accuracy in the training data was achieved at *γ* = 0.7, we used this value in RRF to select features. Similarly, Lasso requires a user-specified regularization parameter (*λ* ∈ [0, 1]), and we determined the optimal value of *λ* =0.03 via grid search (details in Methods). We reported the genes selected by each method in Table 4.

**Table 4:** Genes selected by different approaches

Method		Parameters	Number of Selected Genes	Selected Genes
Lasso		*λ* = 0.03	15	*ATP5J, AURKA, GNPTAB, GPR137B, HSD17B4, IFNGR2, IGFBP5, MED30, MFF, SDC2, SMARCC1, TAF2, TUBB, UBE2J2, ZHX1*
RRF		*γ* = 0.7	13	*ARID4A, CASP3, CAV1, CCND1, CCNH, CDC25C, CDK10, FGF8, IGFBP5, MEN1, MMP3, PDGFB, SEMA3F*
Know-GRRF (*t* = 10, *c* = 0.5)	Cancer gene prior	*δ* = 1	169	Omitted due to space limits.
	Consv. prior	*δ* = 0.5	9	*BCL2L1, BMP4, COL1A1, E2F1, FAS, MEN1, PLAT, RAD23A, TSG101*
	VI prior	*δ* = 0.2	6	*CSF2, DDX6, JUND, MMP3, NOTCH4, PURA*
	All priors	*δ* = 0.5 *β*_*driver*_ = 0.3 *β*_*consv*_ = 0.6 *β*_*vi*_ = 0.1	7	*BMP4, CCNA2, FAS, MEN1, PTPRF, RAD23A, TSG101*

We found that Know-GRRF using priors selected fewer genes than RRF or Lasso using no priors, with the only exception of using cancer-related genes as a single prior. This was likely because a majority of the candidate genes were associated with cancer progression and we assigned the same priority score to all these genes, which lacks the resolution to distinguish one from another.

**Fig. 2:**
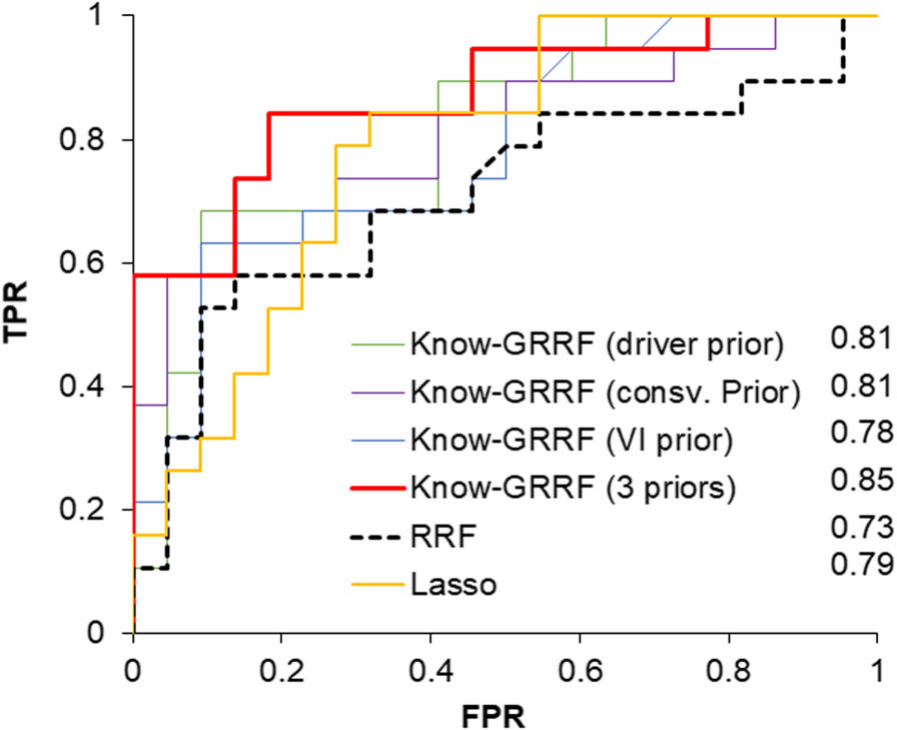
ROC curves of random forests models using genes selected by different approaches. AUROC values are displayed. Data of 41 testing samples were used to construct the curves

With the genes selected by each method, we built a random forests model of 500 trees using the training dataset. We then applied the model to predicting the metastasis status of samples in the testing dataset. We presented the ROC curves and the area under the ROC curve (AUROC) values in Fig. 2. As expected, genes selected by Know-GRRF using priors from all three domains gave rise to the classification model with the highest accuracy (AUROC = 0.85). Conversely, genes selected by RRF had the lowest classification accuracy (AUROC = 0.73). Lasso using no prior reported an AUROC value of 0.79, which was similar to previously reported models for this dataset [7]. Know-GRRF using single-source prior information reported AUROC values ranging from 0.78 to 0.81, implicating that each type of domain knowledge captured the biological relevance of a gene to a certain extent. However, such information was not complete and integrating multi-domain knowledge was helpful.

An advantage of the Know-GRRF algorithm is that the weights (*β* values) can be helpful for interpretation. By examining the weights estimated by Know-GRRF, we found that the conservation prior had the largest contribution to the composite score (*β*_*consv*_ =0.6). This was consistent with previous reports showing mutations and expression changes of evolutionarily conserved genes likely drive the oncogenesis and tumor progression [22].

## Discussion

In high-dimensional data, the number of samples is much smaller than the number of features. This curse of dimensionality causes many features to share similar information gain. When a data-driven algorithm learns a model, it is likely to select a feature that is irrelevant to the classification or regression problem but is associated with another relevant feature by random chance. In these cases, prior knowledge on the relevance of candidate features to the biological question can help eliminate impertinent features and select the truly impactful ones. The fast accumulation and increasing availability of biological knowledge on health phenotypes and quantitative traits offer a great opportunity to employ knowledge-based approaches in biomarker discovery. In this study, we presented the Know-GRRF method that unites the merits of data-driven and knowledge-based approaches and enables the integration of prior knowledge from multiple heterogeneous domains.

Know-GRRF achieves this goal by utilizing a penalty coefficient to regularize the underlying random forests models. Through simulations, we demonstrated that Know-GRRF using multiple complementary prior knowledge was more robust than existing methods that do not use prior information or using incomplete prior information from a single source. Furthermore, Know-GRRF determines the contribution of prior knowledge from each domain in an automated and objective manner. While this is a clear advantage of Know-GRRF, it can potentially introduce a large number of variables for optimization if prior knowledge from many domains on tens of thousands of features need to be combined. In these cases, the computational burden can be prohibitive. Based on our experience in analyzing empirical data, we propose a pre-filtering step using statistical significance to reduce the number of features before running Know-GRRF analysis. For large-scale omics datasets, even using a less-stringent statistical cutoff can remove a majority of uninformative features. We employed this strategy when analyzing the prostate cancer dataset. We showed that the predictive accuracy of genes selected by Know-GRRF was significantly higher than previously reported. We then investigated the seven genes identified by the Know-GRRF model using three types of prior information. All of these genes have been characterized as oncogenes or tumor suppressor genes. In particular, both the *CCNA2* gene and *FAS* gene are activated by the androgen receptor that is the therapeutic target of prostate cancers [23]. Therefore, these gene markers not only provide discriminative power to forecast metastasis, but directly participate in the molecular pathways of prostate cancer progression as well.

It is worth noting that Know-GRRF is not for integrating multi-omics data in a general sense. Instead, data from a specific –omic domain (e.g., whole-exome sequencing or RNA-Seq) needs to be first abstracted into a score for each gene. Then Know-GRRF can use one or more such scores to prioritize genes in a study. On the one hand, this is a limitation of our method. On the other hand, this is an advantage of Know-GRRF to utilize priors from unrelated samples, e.g., sequence conservation during species evolution. Indeed, in our analysis of the prostate cancer microarray data, we defined three sets of independent priors. The first set was derived from literature reviews of multiple gene expression and exome sequencing studies of prostate cancers. The second set was based on sequence conservation of 46 vertebrate species. And the third set was based on variable importance statistics. To our best knowledge, this is the first study that is able to dynamically incorporate such a diverse set of priors for biomarker discovery.

There are some limitations of Know-GRRF. First, features selected by Know-GRRF have some randomness because the algorithm is based on random forests. Although we cannot eliminate bootstrapping completely, we have added a stability selection step to reduce the variation and to increase the reproducibility. Second, the computational cost of Know-GRRF is higher than RRF because it optimizes more variables and builds more ensembles. Third, Know-GRRF does not guarantee global optimization. Thus, several runs with varied initial settings may be required, which further increases the computational cost. Fortunately, these runs are independent from each other and can be executed in parallel. In the future, we will improve Know-GRRF to allow distributed computations.

## Conclusions

In summary, our new method, Know-GRRF is a powerful method to incorporate domain knowledge from multiple resources for feature selection. It has a broad range of applications in biomarker discovery. We implemented this method and released the KnowGRRF package at R/CRAN archive.

## Methods

### Know-GRRF implementation

We have described the algorithm of Know-GRRF in a previous section (see New Method for details). To reiterate, the first component of Know-GRRF aims to find a set of parameters, namely *δ* and *β*_*d*_ within a user-specified range to minimize AIC of OOB samples in a random forests model. For implementation, we used the R packages. Specifically, we wrapped the RRF::RRF () function [17] in the optim () function to perform the BFGS quasi-Newton optimization with box constraints [16]. After the optimal values of *δ* and *β*_*d*_ are determined, we computed the penalty coefficient of each feature according to equations [2, 3]. We then used these penalty coefficients in the second component of Know-GRRF to perform stability selection. After *t* iterations, we took a union of all selected features and used the MASS::stepAIC () function [24] to choose the final set of features.

Feature selection with RRF: We used the RRF package [17]. To determine the best value of the regularization parameter *γ* ∈ [0, 1], we performed grid searching with 20 values equally spaced between 0 and 1. For each value, we selected features and estimated the accuracy in the training set. The value corresponding to the highest training accuracy was taken in the final execution.

Feature selection with Lasso: We used the glmnet package [18]. To determine the best value of the regularization parameter *λ* ∈ [0, 1], we used the glmnet built-in cross-validation function. The *λ* value corresponding to the highest cross-validation accuracy was taken in the final execution.

### Preprocessing of the cancer dataset

We downloaded the gene expression dataset from the NCBI GEO database. The GSE10645 file contains signal values of 1021 probes and the annotation files (GPL5858 and GP5873) mapped the probes to RefSeq genes. For each probe, we used the median value to impute the missing expression data. If multiple probes mapped to the same gene, we took the average expression value of these probes. We then performed z-transformation for each gene to have a distribution with a mean of 0 and a standard deviation of 1 over all samples. These values were subject to further analysis.

### Constructing priors from domain knowledge for the cancer dataset

(1) The cancer gene prior: Nakagawa et al. compiled a list of 526 genes that have been previously associated with prostate cancer progression via literature reviews and previous biomarker studies [20]. We retrieved this list of genes from the annotation file GPL5873 in the GEO database. We then queried the Cancer Gene Consensus [25] and identified 28 driver genes of prostate cancers. Although these 28 driver genes were annotated based on cancer hallmarks and mutational signatures, they are a subset of the 526 genes. We regarded these 526 genes as cancer-related and assigned them a prior score of 10. The other genes received a prior score of 1.

(2) The conservation prior: Given a gene, we first retrieved the multiple alignments of its orthologs in 46 vertebrate species [26] and used the fitch algorithm [27] to compute the absolute substation rate of each position. We then used the average substitution rate over all positions as the evolutionary rate of this gene. Low evolutionary rates indicate high conservation. Therefore, we took the reciprocal of the evolutionary rate as the conservation score of the gene. If a gene had a conservation scores > 10, we reduced it to 10 such that the conservation prior is within the range of 0 and 10.

(3) The variable importance (VI) prior: We first built an RRF model using the training set with the default penalty coefficient value of 0.8. We then retrieved VI values from this model as the priors for Know-GRRF.

## Data Availability

Source codes of simulation, method implementation and identification of prognostic biomarkers are available on Github (https://github.com/guanxin1121/Know_GRRF). Implementation of the method is available as the KnowGRRF package at R/CRAN archive.

## Abbreviations

AIC: Akaike’s Information Criterion
AUROC: Area Under Receiver-Operating Characteristic Curve
FN: False Negatives
FPR: False Positive Rate
GEO: Gene Expression Omnibus
GRRF: Guided Regularized Random Forest
JI: Jaccard Index
Know-GRRF: Know-Guided Regularized Random Forest
OOB: Out-Of-Bag
PSA: Prostate-Specific Antigen
RF: Random Forest
ROC: Receiver-Operating Characteristic
RRF: Regularized Random Forest
TPR: True Positive Rate
VI: Variable Importance
